# Construction of a breeding parent population of *Populus tomentosa* based on SSR genetic distance analysis

**DOI:** 10.1038/s41598-020-74941-w

**Published:** 2020-10-29

**Authors:** Zhiqiang Han, Qiang Han, Yufei Xia, Xining Geng, Kang Du, Jun Yang, Xiangyang Kang

**Affiliations:** 1 The Laboratory of Forestry Genetics, Central South University of Forestry and Technology, Changsha, 410004 Hunan China; 2 Advanced Innovation Center for Tree Breeding by Molecular Design, Beijing Forestry University, Beijing, 100083 China; 3 National Engineering Laboratory for Tree Breeding, Ministry of Education, Beijing Forestry University, Beijing, 100083 China; 4 Key Laboratory for Genetics and Breeding in Forest Trees and Ornamental Plants Ministry of Education, Beijing Forestry University, Beijing, 100083 China

**Keywords:** PCR-based techniques, Plant breeding

## Abstract

Parent selection is the core of hybrid breeding. The breeding strategy involving the parental identification of superior open-pollinated progeny of *Populous tomentosa* germplasm resources can significantly improve the efficiency of parental matching. However, due to some factors such as loose powdering time and pollen competitiveness, the offspring derived from open-pollination families which do not undergo completely random mating. Although hybrid combinations based on the male identification method have a high combining ability, this method cannot easily cover the mating combinations of all male and female specimens in the germplasm bank. In addition, the performance of superior plants in open-pollinated families also affects the selection result. If the trait performance value is higher than the population average, then the special combining ability of the reconstructed hybrid combination may be overestimated. Obtaining a solution to the above problems is of great significance for improving the efficiency and accuracy of selecting hybrid parents of *P. tomentosa*. In this study, 24 pairs of SSR (Simple Sequence Repeats) molecular markers were used to analyze the genetic differentiation of *P. tomentosa* germplasm resources. The results showed that the genetic variation of the *P. tomentosa* population was derived from individuals within the provenance, indicating that high genetic diversity is preserved in provenances. The correlation analysis showed that there was a significant positive correlation between the special combining ability of planting height and diameter at breast height (dbh) of the 34 full-sib progeny population and the genetic distance between the parents. Then, the genetic distance between 18 female plants with high fertility and 68 male plants with large pollen quantity was analyzed using this correlation. Fifteen female parents and 12 male parents were screened out, and 52 hybrid combinations with high specific combining ability for growth traits were predicted. Furthermore, for the male parent identification of superior individual plants, we constructed the breeding parent population including 10 female parents and 5 male parents, generating 14 hybrid combinations with potentially high combining ability. The results of the hybridization test showed that the specific combining ability of plant height and dbh was significantly higher than the controlled pollination. Moreover, genetic distance and paternal identification can be used to rapidly and efficiently construct hybrid parent combinations and breeding parent populations.

## Introduction

The strength of parent combining ability determines the strength of the heterosis and a hybrid combination with strong advantages can only be developed via the breeding of parents with high specific or general combining ability. The traditional selection method relies on a pre-mating design to establish a breeding parent population for the detection and selection of offspring. This method requires considerable time and manpower and physical resources, and the cost is extremely high. Thus, the traditional breeding method has great limitations. Therefore, developing a method to quickly and accurately predict the hybrid parental combination has become a problem with which breeding researchers are extremely concerned and committed to solving.

For the genetic improvement of forest trees, EI-Kassaby et al.^1^ proposed a BWB (breeding without breeding) breeding strategy that uses trees germplasm with a known pedigree to construct a breeding population. This method requires fewer resources than controlled pollination in traditional breeding and has been performed to reconstruct the pedigree of open-pollinated families^1,2^. However, there is a clear sampling error deficiency in the BWB breeding strategy. In other words, the performance of superior individuals in the plantation will affect the selection results. The phenotypic value of the superior plants is higher than the population average, which will lead to overestimation of the special combining ability of the reconstructed hybrid combinations. However, the phenotypic value of superior plants is lower than the population average, which will lead to the underestimation of the special combining ability of reconstructed hybrid combinations. In addition, the generation of open-pollinated families is often affected by many factors, such as paternal pollen competitiveness, flowering consistency, parental location in the group, and wind direction. This is not a completely randomized genetic environment, and certain parents with high combining ability will not have the opportunity to mate to produce offspring. Thus, the hybrid combinations with high combining ability predicted by paternal identification were not comprehensive.

With the development of molecular marker technology as well as techniques to determine the genetic diversity and genetic differentiation of species provenance, new methods of indirectly predicting the combining ability, which improve breeding efficiency, were developed. In crops, Wang et al.^3^ demonstrated that genetic distance can be used as a basis for selecting a superior rice combination. Phumichai et al.^4^ also showed that the genetic distance was significantly positively correlated with the special combining ability of maize yield. Jovan et al.^5^ proved that there was a significant positive correlation between genetic distance and the special combining ability of maize economic traits. In addition, Tian et al.^6^ used molecular markers to analyze the correlation between genetic distance and *Brassica napus* economic traits, and the results showed that there was a significant positive correlation of the two indexes. However, there are exceptions. Perenzin et al.^7^ found that genetic distance has a certain correlation with heterosis in wheat, but the correlation coefficient is small and insufficient to predict heterosis. Dong et al.^8^ analyzed the relationship between genetic distance and heterosis in *Pinus massoniana* and found that genetic distance can be used to predict combining ability and heterosis within a certain genetic distance. The difference in genetic distance and combining ability between the above parents is related to the research material and type of marker applied. Not all molecular markers are suitable for estimating the combining ability between parents. The correlation between genetic distance and combining ability among parents has a close relationship with the research object. Thus, could a positive correlation occur between genetic distance and combining ability in *P. tomentosa*? Can genetic distance be used to predict the high combining ability of hybrid parents to make up for the deficiency of the parent population constructed by paternal identification of open-pollinated superior plants?

In this study, we analyzed the *P. tomentosa* seedlings height and diameter at breast height (dbh) of 34 hybrid combinations and determined the correlation between SSR marker genetic distance and the special combining ability of 19 parentals derived from 5 provenances. The possibility of using genetic distance to predict high combining ability for hybrid combination was discussed, and the open-pollinated individual male identification results were compared with the primary breeding parent population based on genetic distance. Constructing a more reliable high combining ability breeding parent population provides a theoretical reference for overcoming the blindness of hybrid combination and predicting high combining ability combinations of *P. tomentosa*.

## Materials and methods

### Materials

**Figure 1:**
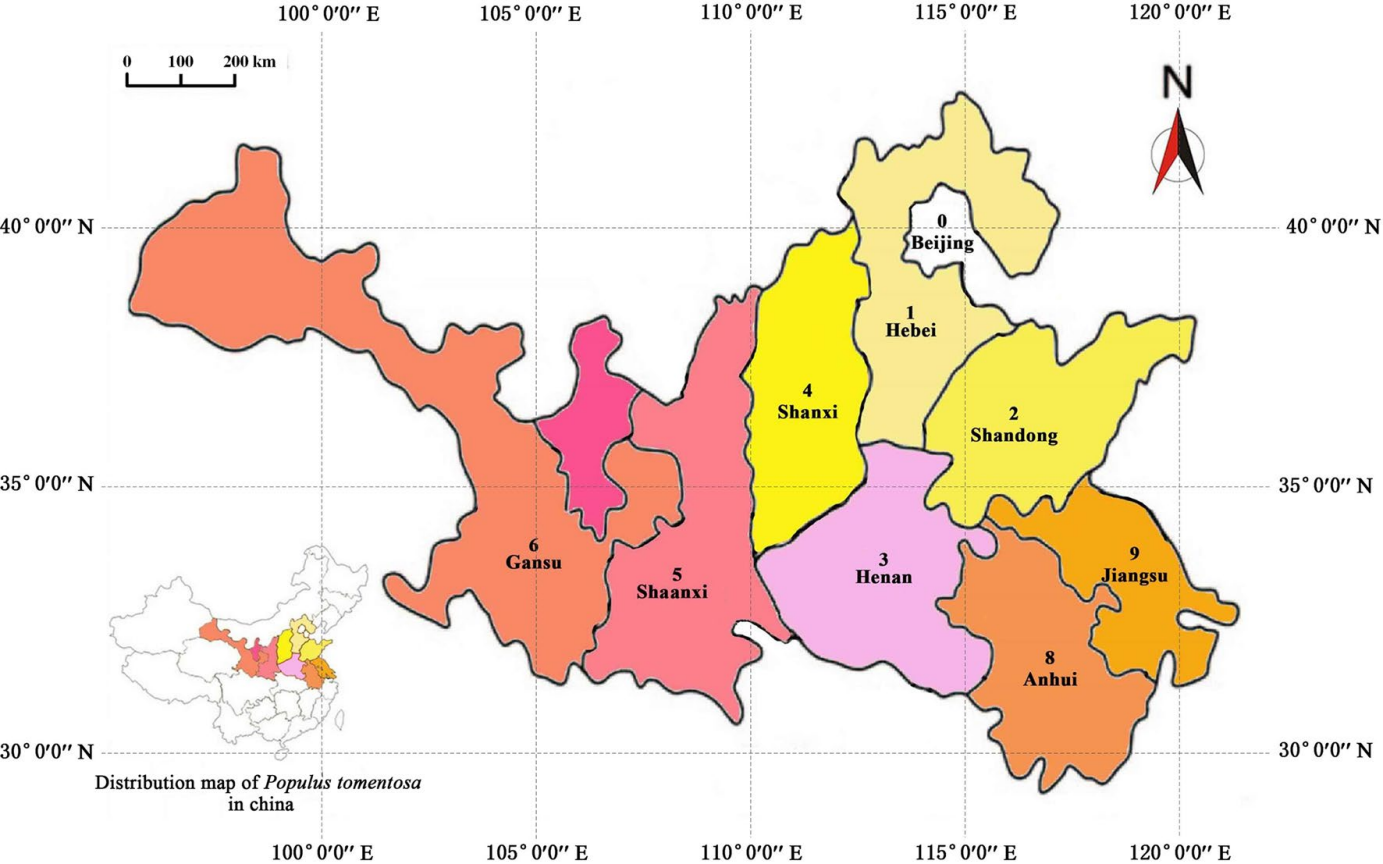
Locations of the nine sampled populations are shown by patterns in different colors, and the thin black lines represent the meridian and parallel. The locations of the 12 distribution areas are represented by red dots.

From 1983 to 1984, the *P. tomentosa* Research Institute of Beijing Forestry University (China, Beijing) organized the provincial *P. tomentosa* research collaboration organization to carry out a preliminary resource survey. In the Huanghe-Huaihe-Haihe river basin, the *P. tomentosa* distribution area is 1 million km^2^ (30–40° N, 105–125° E) in 100 counties, and 1047 high-quality *P. tomentosa* trees were selected. In 1986, the germplasm resources bank of *P. tomentosa* in Guan Xian County of Shandong Province was established. At present, 441 genotypes of excellent diploid trees from Beijing, Hebei, Shandong, Henan, Shanxi, Shaanxi, Gansu, Anhui, and Jiangsu provinces are preserved in the germplasm bank, 18 of which have been screened for good fertility and high combining ability of the female plants^9^, and 68 male plants with large pollen quantities were also identified^10^. The female plants are numbered T-F-1 to T-F-18, and the male plants are numbered T-M-1 to T-M-68. In addition, 17 female plants and 2 male plants distributed in 11 counties (Fig. 1) were selected, 34 hybrid combinations (Supplementary Table S1) were constructed by cross-matching of test lines, and 980 seedlings were obtained. Furthermore, 3984 seedlings from eight full-sib hybrid progeny populations, used for hybridization verification, were planted in the nursery of Guan Xian County, Liaocheng City, Shandong Province.

### DNA extraction and SSR analysis

Total DNA extraction was performed using a plant genomic DNA extraction kit (Tiangen Biotech Co. Ltd, Beijing, China). In this study, three types of primers were required according to Schuelke's method^11^, including forward primers with a 5′-terminus M13 sequence primer (5′-TGTAAAACGACGGCCAGT-3′), common reverse primers, and fluorescent (ROX, FAM, TAMRA, and HEX) M13 primers. The specific PCR procedure is as follows: 94 °C for 5 min; 94 °C for 30 s; 25 cycles of 72 °C for 30 s; 94 °C for 30 s, 53 °C for 30 s, 72 °C for 30 s, 8 cycles; and 72 °C for 8 min. The products were then stored at 4 °C. The PCR products were generated by Ruibo Xingke Biotechnology (Beijing) Co., Ltd. on an ABI-3730XL Genetic Analyzer, and the results were read and analyzed using GeneMarker 1.75 software^12^.

### Genetic analysis of different provenances

A total of 536 pairs of SSR primers in the SSR database (IPGC; httpa/w.ornl.gov/sci/ipgc/ssr resource.htm) published by the International Populus Genome Consortium were randomly selected as screening objects. All the forward primers were labeled with M13 fluorescent dye at the 5′ tail end, and all the primers (forward primer, reverse primer and M13 fluorescent labeling primer) were entrusted to Beijing Ruibo Xingke Biotechnology Co., Ltd. for synthesis. The above primers were used for TP-MI3-SSR PCR to screen for SSR polymorphism primers with differences among provenances.

The original data were converted as needed using CONVERT VERSION 1.3.1 software^13^. The number of allele loci (*N*_*A*_), effective allele number (*N*_*E*_), expected heterozygosity (*H*_*E*_), observed heterozygosity (*H*_*o*_) and Shannon's polymorphism index (I) were calculated by software POPGEN Version 1.32^14^ for all clones of the *P. tomentosa* populations. These parameters can ideally reflect the genetic diversity of the population as a whole and sub-regions. The F-statistic index (*F*_*IS*_*, F*_*IT*_*, F*_*ST*_)^15,16^ was estimated by FSTAT 2.9.3 software^17^, where the genetic differentiation coefficient (*Fst*) reflects the degree of the population genetic differentiation. The fixation indices *F*_*IT*_ and *F*_*IS*_ represent degree of inbreeding at the total population level and between individuals in each subpopulation, respectively^18^. The estimated allele frequencies were imputed by GenAlEx version 6.5^19^ software to compute the average value across markers of the standardized genetic differentiation measure (Gst) proposed and recommended for SSR data by Hedrick^20^.

Polymorphism information content (PIC) was calculated using PIC-CalcVersion 0.6 software to evaluate genetic diversity^21^. Rousset's genetic distance model $\frac{{F}_{ST}}{1-{F}_{ST}}$ was used to estimate the genetic distance between individuals within provenances^22^. The geographical distance was based on the latitude and longitude of the sampling point, and Vincenty's formula (https://www.movable-type.co.uk/scripts/latlongvincenty.html) was used to calculate the geographic distance (km) between the provenance and the individuals within the provenance. In addition, the Distance Web Service (Version 3.23) was used to perform a Mantel correlation test of genetic distance and geographic distance^23,24^.

### Estimation of genetic parameters

The height and diameter at breast height data for 34 full-sib families (Table 1) were obtained by measuring for three consecutive years. The SPSS software was used to calculate the relevant parameters of all phenotypic traits. The genetic parameters analysis (VSN International, Hemel Hampstead, UK) was performed using ASReml-R 4 software^25^, and a Pearson correlation analysis was performed using Origin pro version 9.0. There were F families, with the observed value of a single plant as the statistical unit. Tree height and diameter at breast height data were analyzed by the following mixed linear model for restricted maximum likelihood analysis: ${\mathrm{y}}_{ij}=\upmu +{M}_{i}+{F}_{j}+\left({MF}_{ij}\right)+{e}_{ij}$, where y_*ij*_ is the observation of the *i*-th male parent and the *j*-th female parent, μ is the universal mean, and e_*ij*_ is the residual effect.

**Table 1:** *G*_ST_ between the nine native *P. tomentosa* populations based on 24 SSR markers.

	Beijing	Hebei	Shandong	Henan	Shanxi	Shaanxi	Gansu	Anhui	Jiangsu
Beijing									
Hebei	0.000								
Shandong	0.003	0.004							
Henan	0.029	0.029	0.016						
Shanxi	0.000	0.000	0.002	0.027					
Shaanxi	0.045	0.046	0.032	0.012	0.042				
Gansu	0.002	0.005	0.004	0.011	0.004	0.017			
Anhui	0.002	0.000	− 0.002	0.007	0.000	0.022	− 0.001		
Jiangsu	− 0.002	− 0.002	− 0.003	0.013	− 0.003	0.033	− 0.003	− 0.007	

*G*_ST_: equivalent to F_ST_ but estimator with different statistical properties.

The random effects of genetic variance include:

- *F*i, which is the random effect of general combining ability of the parent i, E(Fi) = 0, Var(Fi) = σ^2f^;
- *M*j, which is the random effect of general combining ability of the father j, E(Mj) = 0, Var(Mj) = *σ*^2^_*m*_;
- *FM*_ij_, which is the special combining ability random interaction effect of female parent *i* and male parent *j*, E (*FM*_ij_) = 0; Var(FMij) = *σ*^2^_*fm*_^26–28^.

## Results

### Genetic differentiation among provenances of *P. tomentosa* germplasm resources

Genetic differentiation was performed at 24 polymorphic SSR loci (Supplementary Table S2). There were significant genetic differentiation levels in various provenances (P < 0.01). The PIC parameters varied from 0.232 (Jiangsu) to 0.461 (Henan), with an average of 0.332, indicating a lower level of allelic diversity in the *P. tomentosa* germplasm resource bank (Supplementary Table S3).

In the genetic analysis of the germplasm resources from the nine provenances, we found that the mean fixation index within provenance (*F*is) was negative in the nine provenances, ranging from − 1.0 to − 0.237 (Supplementary Table S3). The genetic differentiation ranged from 0.031–0.036. The genetic differentiation was low between pairwise provenances, varying from − 0.007 to 0.046, indicating that the genetic diversity is distributed within provenances (Table 1). The differences among individuals within the population were the main sources of genetic variation, indicating that higher genetic diversity was preserved within the provenance.

### Analysis of combining ability of plant height and diameter at breast height of full-sib hybrid combination

The specific combining ability for tree height and diameter at breast height of 34 hybrids combinations was analyzed (Table 2). The results showed that the maternal or paternal effect of different provenances had no significant effect on the height and diameter at breast height of the progenies. The difference between height and diameter at breast height between the hybrid combinations reached a very significant level (P < 0.01), indicating that the parental combination has a very significant effect on the special combining ability of height and diameter at breast height.

**Table 2:** Variance analysis of specific combining ability values in seedling height and diameter at breast height.

Phenotypic traits	Variation sources	DF	MS	Random model	
				F value	P value
Seedling height	Male	1	112.76	1.90	0.168
	Female	16	167.94	2.84	< 0.01**
	Male × female	16	66.74	1.13	< 0.05*
	Error	646	59.20		
Diameter at breast height	Male	1	3.15	0.08	0.778
	Female	16	189.44	4.82	< 0.01**
	Male × female	16	81.04	2.06	< 0.01**
	Error	646	39.30		

**Figure 2:**
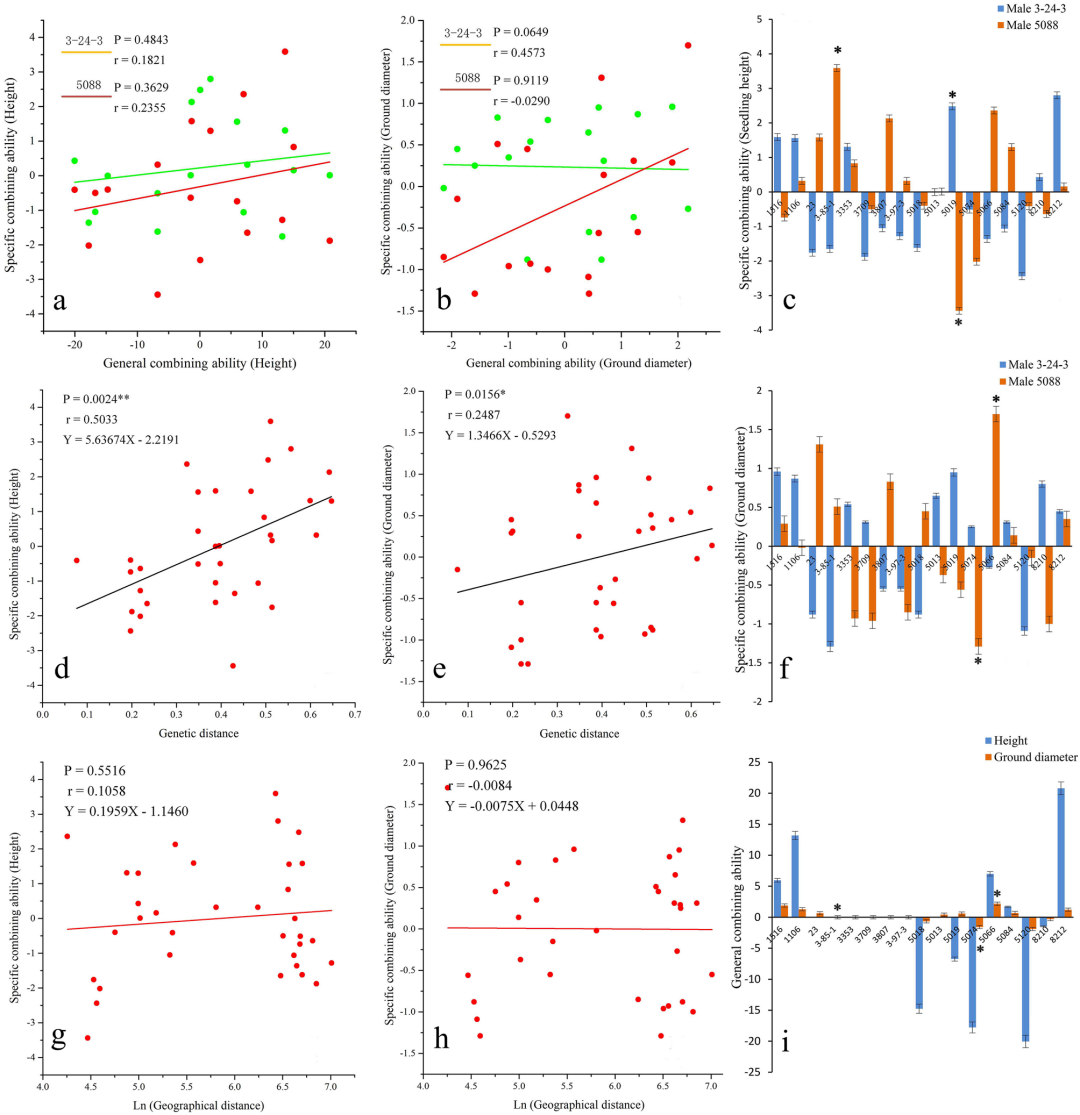
Relationship among the general combining ability, specific combining ability, genetic distance and geographical distance for height and ground diameter in *P. tomentosa*, * and ** indicate the 0.05 and 0.01 significance level, respectively.

The results showed that among the 34 hybrid combinations, the special height combining ability effect value of the combination 3-85-1 × 5088 was the largest, and the height general combining ability effect value of the female parent 3-85-1 was the lowest (Fig. 2i). The special combining ability effect value of height special combining ability of 5019 × 5088 was the smallest (Fig. 2c), while the height general combining ability effect value of the female parent 5019 was generally larger (Fig. 2i), and a correlation with the special combining ability was not observed (Fig. 2a). The special combining ability of the diameter at breast height of 5066 × 5088 was the largest, and the general combining ability effect value of the diameter at breast height of 5066 was also the largest (Fig. 2f,i). The special combining ability of the diameter at breast height of the combination of 5074 × 5088 was the smallest, and the general combining ability effect value of the diameter at breast height of the 5074 was close to the lowest (Fig. 2f,i).

Pearson correlations between height and diameter at breast height general combining ability and the special combining ability of different provenance parents were analyzed (Fig. 2a,b). The height general combining ability of different provenance parents was positively correlated with the special combining ability. The hybrid fathers 3-24-3 and 5088 had correlation coefficients of 0.1821 and 0.235, respectively, but did not reach a very significant correlation level. However, the diameter at breast height general combining ability value and the special combining ability effect value of different provenance parents showed different correlation trends. For hybrid parent 3-24-3, the general combining ability of diameter at breast height was negatively correlated with the special combining ability. The correlation coefficient was − 0.029, and the correlation did not reach a significant level. For the hybrid parent 5088, the general combining ability of the diameter at breast height was positively correlated with the special combining ability, and the correlation coefficient was 0.457. This correlation also did not reach a significant level.

The genetic distance and geographical distance between each parental individual were estimated (Supplementary Table S4; Fig. 2d,e), and the special combining ability effect of the height and diameter at breast height of hybrid combination and its corresponding inter-individual geographical distance were shown by the Mantel test (Fig. 2g,h). The height of each parental combination was positively correlated with the geographical distance, and the correlation coefficient was 0.106, which did not reach a significant level (P = 0.5516). The diameter at breast height of each parental was not significantly (P = 0.9625) correlated with the geographical distance (r = − 0.008). Thus, using the geographical distance to predict the special combining ability is not possible. A correlation analysis between height and diameter at breast height special combining ability and the corresponding genetic distance showed that the height and diameter at breast height were positively correlated with the genetic distance (P = 0.0024; P = 0.0156). Thus, the genetic distance had a very significant effect on the special combining ability of height and diameter at breast height, respectively.

### Prediction of hybrid parental population with high special combining ability of *P. tomentosa*

The relationship between the special combining ability and the genetic distance was verified, and the genetic distance was positively correlated with the special combining ability, which indicates that genetic distance can be used to predict the breeding parents. Due to the serious abortion problem in *P. tomentosa*, the reproductive ability was weak, and many female parents are not suitable for use in hybridization by artificial pollination. Therefore, the selection of parents should be a male and female with a certain fertility and a large genetic distance. Based on the above principles and previous research, 18 females with high fertility and high general combining ability and 68 male trees with large pollen quantities were selected from the excellent tree germplasm resources of *P. tomentosa* to construct a primary breeding parent population with potentially high special combining ability. The parental genetic distance of 1224 hybrid combinations (Supplementary Table S5) ranged from 0.032 and 0.984, with an average of 0.424 (Table 3). A genetic distance greater than 0.710 (i.e., the genetic distance average + 2 standard deviations) was used as the standard for the construction of the primary breeding parent population. There are 52 hybrid breeding combinations with a large genetic distance, including 15 female parents and 12 male parents (Table 3).

**Table 3:** Prediction of the parent group with high specific combining ability in *P. tomentosa*.

	Male	Female	Max	Min	Mean	SD	High specific combining parents	
Parent populations	18	68					♀:15	♂:12
Genetic distance			0.984	0.032	0.424	0.143		
Hybrid combination	1224						52	

♀ represents the female parent, ♂ represents the male parent, and SD represents standard deviation.

The greatest number of hybrid combinations with high specific combining ability for male plants T-M-14, T-M-31, and T-M-45 was 13, 13, and 14, respectively (Supplementary Table S7). In addition, male plants T-M-43 and T-M-35 participated in the combination with high specific combining ability twice, while male plants T-M-2, T-M-17, T-M-41, T-M-44, T-M-58T-M-59 and T-M-61 participated in the combination with high special combining ability for the least number of times (all one time). At the same time, the female plants T-F-6, T-F-7, T-F-14, and T-F-17 were more frequently used in the combination with high specific combining ability, at 8, 6, 4, and 4 times, respectively. However, the high specific combining ability hybrid combinations for female plants T-F-1, T-F-3, T-F-4, T-F-5, T-F-8, T-F-9, T-F-13, T-F-15 and T-F-16 were used 3 times, and the fathers were T-M-14, T-M-31, and T-M-45. In addition, T-F-18 was involved in the high specific combining ability hybrid combination with the least number of times (one time).

### Parental selection for high combining ability based on the genetic distance and male parent identification of open-pollinated superior plants

**Figure 3:**
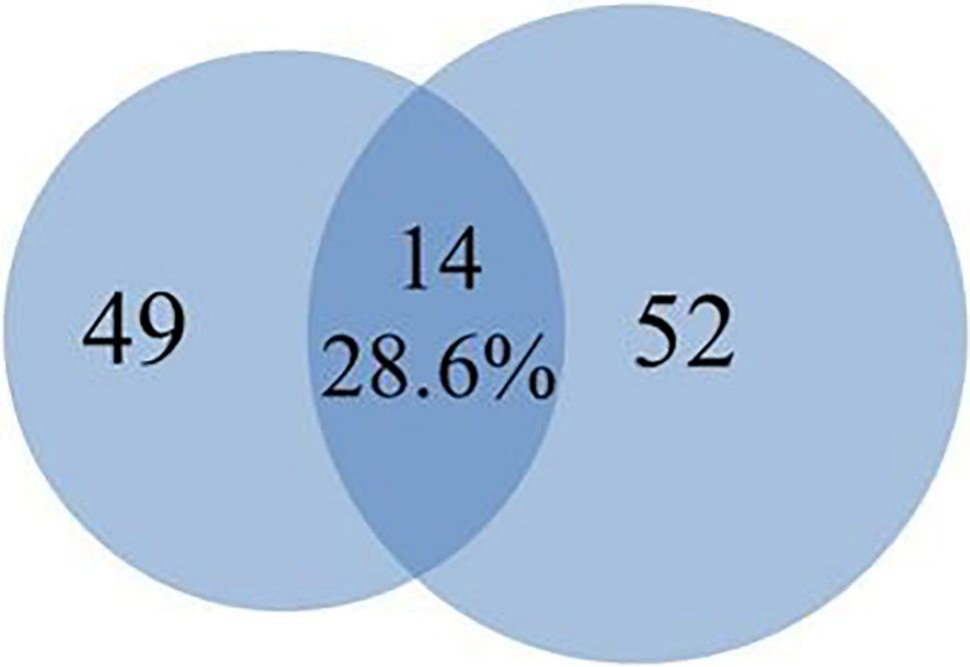
Comparison of hybrid combinations predicted by genetic distance and paternal identification.

The number of hybrid combinations for the genetic distance predictions and male parent identification of the open-pollinated excellent trees were 52 and 49 (Supplementary Table S7,8), respectively. According to the hybrid combinations predicted by genetic distance, the hybridization combination was not the same as that of the open-pollinated individuals parent identification (Fig. 3). Among the 52 hybrid combinations predicted by genetic distance, only 14 hybrid combinations were consistent with the paternal identification, and the parent identification group accounted for 28.6% of the hybrid combination. The 35 hybrid combinations identified by the open-pollinated individuals parental identification were not found in the high specific combining ability hybrid combinations predicted by genetic distance.

The genetic distance and superior open-pollinated individuals parent identification predicted 14 high specific combining ability hybrid combinations, including 10 female (T-F-1, T-F-3, T-F-6, T-F-7, T-F-8, T-F-9, T-F-14, T-F-15, T-F-16, and T-F-18) and 4 male (T-M-14, T-M -43, T-M-45, and T-M-61) parents (Table 4).

**Table 4:** Statistical table of high specific hybridization combinations based on parents’ genetic distance and half-sib progeny paternal identification.

Male	Female									
	T-F-1	T-F-3	T-F-6	T-F-7	T-F-8	T-F-9	T-F-14	T-F-15	T-F-16	T-F-18
T-M-14	○	○	○/□		○	○	○	○/□	○	
T-M-31	○	○	○		○	○	○	○	○	
T-M-43				○			○/□			□
T-M-45	○/□	○/□	○/□	○/□	○/□	○/□	○/□	○/□	○/□	○/□
T-M-61			○/□							

○ represent high specific hybridization combination based on genetic distance between parents.

□ represents hybridization combinations based on the identification of the male parent.

○/□ represents hybrid combination predicted based on the above two methods.

### Hybridization verification of parents based on genetic distance and parent identification

Referring to the genetic distance and the paternal identification results for the open-pollinated excellent plants, three female (T-F-15, T-F-14, T-F-18) and six male (male T-M-14, T-M-45, T-M-43, T-M-2, T-M-27, T-M-41) parents were randomly selected. In February 2016, 8 full-sib hybrids populations were constructed, and the effectiveness of hybridization of parents based on genetic distance and parental identification of superior plants was verified. Among them, four hybrid combinations based on genetic distance and parental identification of superior plants were selected: T-F-15 × T-M-14, T-F-14 × T-M-45, T-F-14 × T-M-43, and T-F-18 × T-M-45. Only T-F-15 × T-M-41 and T-F-18 × T-M-43 were predicted by paternal identification. The two prediction methods predicted T-F-15 × T-M-2 and T-F-14 × T-M-27. Through the construction of the full-sib hybrid progeny population, a total of 34,020 hybrid seeds were obtained, 5415 full-sib child progeny hybrid seedlings were obtained, and 3984 seedlings were obtained. The emergence rate was 15.9%, and the survival rate was 73.6% (Supplementary Table S9).

Height and diameter at breast height were measured for the full-sib hybrid progeny population (Table 5). The average value in height of the eight hybrid combination progenies is 193.85 cm, and the average value in diameter at breast height is 17.06 mm. The combination of T-F-14 × T-M-43 has the highest height and diameter at breast height, which are 250.67 cm and 22.68 mm, respectively. The combination T-F-14 × T-M-45 has the second highest height and diameter at breast height, and the two hybrid combinations have higher height and diameter at breast height than the control combination of the same female parent TF-14 × TM-27. The same result is obtained in the hybridization with T-F-15 and T-F-18 as the female parent.

**Table 5:** Analysis of variation and special combining ability of seedling height and diameter at breast height for different *P. tomentosa* hybrid combinations.

Hybrid combination	Height (cm)	Amplitude of variation (cm)	CV (%)	SCA	Diameter at breast height (mm)	Amplitude of variation (mm)	CV (%)	SCA
T-F-15 × T-M-2	138 ± 53.4 e	70–360	38.7	− 5.3	14.22 ± 5.5 c	7–32	38.7	− 2.6
T-F-15 × T-M-14	232.35 ± 53.7 a	140–400	23.1	3.8	18.84 ± 7.2 b	6–34	38.0	1.7
T-F-15 × T-M-41	186.08 ± 68.1 c	50–370	36.6	− 0.6	17.05 ± 6.1 b	5–32	35.6	0.01
T-F-14 × T-M-27	152.33 ± 45.8 e	60–280	30.0	− 3.8	14.11 ± 5.0 c	5–28	35.3	− 2.7
T-F-14 × T-M-45	210.66 ± 56.8 b	100–390	27.0	1.7	17.79 ± 4.1 b	9–30	22.8	0.7
T-F-14 × T-M-43	250.67 ± 70.3 a	110–410	28.0	5.5	22.68 ± 6.0 a	11–33	26.5	5.1
T-F-18 × T-M-43	161.61 ± 61.1 d	30–290	37.8	− 3.0	14.59 ± 5.7 c	5–29	39.1	− 2.3
T-F-18 × T-M-45	209.04 ± 54.7 b	100–400	26.2	1.6	17.09 ± 6.3 b	7–34	36.7	0.04
	193.85 ± 71.2	90–410			17.06 ± 6.3	4–27		

Note: Different lowercase letters represent difference at 0.05 significance level, CV = Coefficient of variation, and SCA = Specific combining ability.

The combining ability analysis of growth traits by ASReml-R 4 software showed that the special combining ability of height and diameter at breast height of the hybrid combinations T-F-14 × T-M-45 and T-F-14 × T-M-43 was significantly higher than that of the same female parent. In the hybrid combination with T-F-18 and T-F-15 as the female parent, the special combining ability of plant height and diameter at breast height was also significantly higher than that of the control hybrid combination. There were large variations in height and diameter at breast height of the hybrid progeny in each hybrid combination. The variation coefficient of height ranged from 23.1 to 38.7%, and the variation coefficient of diameter at breast height ranged from 22.8 to 39.1% (Table 5). The hybrid combinations T-F-18 × T-M-43 and T-F-15 × T-M-41 were only predicted by the paternal identification method, and the hybrid combinations T-F-15 × T-M-14, T-F-14 × T-M-45, T-F-14 × T-M-43 and T-F-18 × T-M-45 were predicted by the paternal identification and genetic distance methods (Table 4). As displayed in Table 5, The paternal identification and genetic distance methods jointly predicted that the seedling height and diameter at breast height measurements of the hybrid combinations and their special combining ability values would be higher than those of the other hybrid combinations of the same female parent. However, the height, diameter at breast height and special combining ability of hybrid combinations T-F-15 × T-M-41 and T-F-18 × T-M-43 predicted by paternal identification were lower than those predicted by paternal identification and genetic distance. The height and diameter at breast height of the hybrid combination T-F-15 × T-M-41 were higher than those of the T-F-15 × T-M-2 hybrid combination. T-F-15 × T-M-2 was the dominant hybrid combination, and it was not predicted by the combination of parental identification and genetic distance prediction. The paternal identification method has a certain ability to predict strong dominant hybrid combinations, although the joint prediction by the paternal identification and genetic distance method is more effective than paternal identification alone.

## Discussion

### Genetic variation analysis among provenances

In this study, 24 pairs of SSR primers were used in the genetic analysis of 441 clones of *P. tomentosa* other than the natural triploid of *P. tomentosa*. The results show that there is excess of heterozygosity within provenance (*F*_IS_ = − 0.540) and low genetic differentiation among *P. tomentosa* provenance (*F*_ST_ = 0.037), which may be due to the long-term cultivation and introduction of *P. tomentosa*. The same pattern of genetic variation was also found in close relatives of *P. tomentosa*, *Populus tremula*^29–31^. To carry out the genetic improvements of *P. tomentosa*, individual differences within the population should be considered, and hybrid parents with large genetic distances should be selected to acquire high heterosis. A correlation analysis between individual genetic distance and geographical distance revealed that the geographic distance is not important. Furthermore, the 34 full-sib hybrid family derived from the same provenances or different provenances are analyzed. The results show that the genetic distance between the parents is significantly positively correlated with the special combining ability of the full-sib growth traits, and a larger genetic distance corresponds to strong heterosis. Therefore, the analysis of the correlation between the genetic distance and the special combining ability in *P. tomentosa* has important guiding significance for the selection of hybrid combinations. In the *P. tomentosa* population, the breeding strategy in which strong heterosis combinations are selected based on the parents genetic distance can be used to significantly reduce the selection range of parents, thus saving breeding time and a large amount of human and material resources; moreover, it lays the foundation for the rapid and efficient construction of high combining ability hybrid parent groups.

### Parent selection for breeding based on genetic analyses

The general combining ability reflects the average performance of the progenies resulting from several mating combinations in a mating group and reacts to the parental additive gene effect, and the additive gene can be transmitted to the offspring in the mating population. The special combining ability reveals the deviation between the performance of a particular mating combination and the predicted performance of the parent general combining ability. The additive effect and the superior effect of the reactive gene can only be expressed when specific genes are combined and cannot be passed on to the offspring^32^. In this study, a significant correlation is not observed between the general combining ability and special combining ability of height and diameter at breast height. This relationship has been reported in recent research^33,34^. The relationship between the special combining ability and the genetic distance was verified, and the genetic distance in *P. tomentosa* was significantly positively correlated with the special combining ability, indicating that the breeding parents could be predicted by the genetic distance. However, Tian et al. used the genetic distance of rapeseed economic traits to predict heterosis and combining ability and found that there was no significant correlation between the two indices^6^, indicating that the genetic distance is not capable of predicting heterosis and combining ability^35–40^. This finding may be related to the lack of a relationship between the determinant gene of the measured trait and the molecular marker utilized to estimate the genetic distance or differences between individual genomes of the offspring and interactions between genes and the environment^41–44^, thus illustrating the complexity of quality traits^36^.

Based on the genetic distance analysis, a primary breeding parent population with potentially high specific combining ability was constructed, and the high specific combining ability hybrid combination was predicted; however, only 14 hybrids were consistent with the results of the paternal identification of open-pollinated individuals. The reason may be related to the inconsistency of the loose powder time of the male plants or the difference in the competitiveness of the pollen, meaning that the parent population identified based on open-pollinated excellent plants is not obtained under a genetic environment with completely random mating. However, 35 of the reconstructed hybrid combinations based on the parental identification were different from those with high special combining ability predicted by genetic distance, which may be due to the influence of random sampling error. Therefore, the selected superior plants were the most prominent individuals in the progeny population, and the average value of the growth traits of the full-sib population was relatively low, which may also explain why the male plant T-M-31 can be combined with 13 high fertility and high combination ability female plants as a high special combining ability hybrid combination via the genetic distance prediction but not by the parental identification method^45^. Here, we included T-M31 in the breeding parent population, which was the final constructed breeding parent group that included 10 female parents and 5 male parents. The full-sib offspring representative assay was used to verify that height and diameter at breast height of the predicted high specific combining ability hybrid combination were significantly higher than those of the same female parental control hybrid combination. The results showed that the genetic distance and the paternal identification results of the half-sib progeny are able to quickly and effectively construct breeding parent groups and confirm breeding parents.

### Forest tree breeding parent matching strategy

In forest genetic improvement, BWB strategies have effectively improved breeding efficiency^2^. However, there are obvious shortcomings in the BWB breeding strategy, such as sampling error and incomplete randomization of the mating genetic environment, which may result in the selection of superior trees that do not represent the comprehensive performance of the progeny population of elite hybrid combinations. This study combines genetic distance and BWB breeding strategies to improve the accuracy and effectiveness of parental selection and breeding parent population construction. Based on the above research, this paper proposes a breeding parent selection strategy based on a high special combining ability. For the selection of excellent *P. tomentosa* plants and even similar species, the germplasm resource bank is first used to observe and screen parents with good fertility through pollen and seed formation. Molecular markers are used to analyze the genetic distance of the fertile parents. Based on certain genetic distance criteria, the parental hybrids with potential high specific combining ability are screened to construct the primary breeding parent population. Furthermore, the open-pollinated family is constructed by using the female parents of the primary breeding parental group, excellent offspring trees are screened from this group, and the paternal identification of open-pollinated excellent individuals is analyzed by molecular markers. On this basis, a primary breeding parental group and parent identification results are synthesized using the genetic distance to select parents that match the high special combining ability hybrid combination.

## Supplementary information

Supplementary Information.
