# Supplementary material for: Construction of a breeding parent population of Populus tomentosa based on SSR genetic distance analysis
Source: Sci Rep. 2020 Oct 29;10:18573. doi: 10.1038/s41598-020-74941-w (PMC7596703; doi:10.1038/s41598-020-74941-w)
Supplement: Supplementary file 1 — Supplementary Information. [file 41598_2020_74941_MOESM1_ESM.pdf]

Construction of a breeding parent population of *Populus tomentosa* based on SSR genetic distance analysis

Zhiqiang Han·Qiang Han·Yufei Xia· Xining Geng·Kang Du· Jun Yang·Xiangyang Kang

Supplemental material

Table S1 Tester strain mating design among 2 male and 17 female parents for *P. tomentosa*

Male	Female																
	1516	1106	2-3	1417	3353	3709	3807	4128	5018	5013	5019	5074	5066	5084	5120	8210	8212
3-24-3	*	*	*	*	*	*	*	*	*	*	*	*	*	*	*	*	*
5088	*	*	*	*	*	*	*	*	*	*	*	*	*	*	*	*	*

Table S2 Sequence of primers used in present study

Marker	Primer sequence (5′-3′)	Repeat motif
LG_III-2	Forward: ATTGATTATATTTGCCGCAT<br>Reverse: TGGACATCTCACTACCTTCC	AT
GCPM_2570-1	Forward: AACCCACTTCCTCTCTCTGT<br>Reverse: TGAGACTTCCGACTCGTAG	CT
PMGC_2606	Forward: AATTTACATTTCTTTATCATCACC<br>Reverse: GCTGTCTAACATGCCATTGC	GA
ORPM_197	Forward: GTCAGTTTGCCCTCTTCGTC<br>Reverse: TGAGGGCGTCTCCTCTTTTA	GA
PMGC_2140	Forward: GCTGTCAGAATCAAACACTTC<br>Reverse: AAGCAGATAACTAAGACATGCC	GA
PMGC_223	Forward:CGATGAGGTTGAAGAAGTCG<br>Reverse: ATATATGTACCGGCACGCCAC	CTT
LG_IX-1	Forward: CCAACTTCAATGCTAGGAAC<br>Reverse: TGGTAAGCCTGAGGATACAC	AG
LG_VIII-3	Forward: ATCCGACTTCGATATCTTCA<br>Reverse: CTACCTGAAACACAGGAAGC	CT
GCPM_112-1	Forward: TTAGAGGAGAGAACTGCTGC<br>Reverse: TGGTCTGCAACACAAGATT	GT
GCPM_2627-1	Forward: TAAGTCCCACTACACCCAAC<br>Reverse: GAGTTCGAGAGAGGGAATCT	CAC
LG_VIII-4	Forward: ATCTTTCAAGAAGCTTGACG<br>Reverse: AGCATTCTTGCTGGTGTTAT	AG
GCPM_1504-1	Forward: AGGTCTGTGCAAGGAATAAA<br>Reverse: GTCTGTAATCAAGCCAAAGC	TC
GCPM_1411-1	Forward: TCAACGACTTTTTCATTGTG<br>Reverse: AGCATTCTTGCTGGTGTTAT	TGC
LG_V-2	Forward: AAAGAAACCAGACCACACAC<br>Reverse: CGCTTGCCTTAATTAACAGT	TC
GCPM_1153-1	Forward: TTCCTTTCACACAATGACAA<br>Reverse: TTTAAAAACTGGGTCCGTAA	CTT
GCPM_1524-1	Forward:TTCAATGGAAAGGGATAATG<br>Reverse: TCATTTGTAAAACATCACGC	AAC
LG_XVI-6	Forward:ATAGCGATCATCAAAGGAAA<br>Reverse: AAATATTCATGTGGAGGCAC	ATG
LG_XVI-9	Forward:CTCGCAGCTCTTCTCATAGT<br>Reverse: CCTACCCATTTATGACCAAA	TGC
PMGC_2818	Forward: AAGCTTCATCGTCCTGCTTG<br>Reverse: CGTATCAATTCACGACTCTCG	GA
GCPM_2768	Forward: CAGCAAAATCATCACAAATC<br>Reverse: AGGGTTTGGTAGAGAAGACC	GA
LG_XVI-7	Forward: ACAAATCAAAGTCACAGCCT<br>Reverse: ATAGTGTTCAATCGGACCTG	AATC
GCPM-2012	Forward: GGTGATGAAGATCTGGGATA<br>Reverse: ACCCAAATTACAGAACAACG	ATA
GCPM_1832-1	Forward: TTACTTGCTAGCTGCCAATC<br>Reverse: CCTAAAAGTTTGTCTATGCGA	TA
GCPM_3367-1	Forward: CAGGACATTTAACTCCTTCG<br>Reverse: TTAAGTCCTCAACAGAGGGA	GCC

Table S3. Genetic diversity statistics for 24 SSR loci in 9 provenances

				Heterozygosity			*F*-statistics			P-Value
	N_A	N_E	I	PIC	H_O	H_E	F_{IS}	F_{IT}	F_{ST}	
Beijing	2.25	1.70	0.5319	0.246	0.6425	0.3633	-0.8241	-0.8166	0.03531	0.0001
Hebei	3.75	1.73	0.5803	0.288	0.6401	0.3613	-0.7591	-0.7570	0.03509	0.0015
Shandong	3.58	1.91	0.7429	0.364	0.6491	0.4446	-0.5242	-0.5003	0.03307	0.0004
Henan	4.96	2.30	0.9416	0.461	0.6382	0.5197	-0.2372	-0.2338	0.03068	0.0035
Shanxi	3.50	1.74	0.5951	0.293	0.6384	0.3719	-0.7191	-0.7263	0.03486	0.0076
Shaanxi	4.04	2.28	0.9040	0.453	0.6837	0.5231	-0.3358	-0.3165	0.03068	0.0000
Gansu	2.25	1.81	0.3045	0.317	0.6354	0.4181	-0.6661	-0.6343	0.03423	0.0086
Anhui	2.42	1.88	0.6493	0.337	0.6369	0.4382	-0.6168	-0.5443	0.03381	0.0001
Jiangsu	1.71	1.67	0.4597	0.232	0.6250	0.3861	-1.0000	-0.9367	0.03622	0.0023

Note: N_A = Number of alleles per locus; N_E = Effective number of alleles; I = Shannon's Information index; H_O = Observed heterozygosity; H_E = Expected heterozygosity; F =Wright's fixation index; PIC= Polymorphism information content; F_{IS} = Inbreeding among individuals within subpopulations; F_{IT} = Inbreeding within entire population; F_{ST} = Variation due to differentiation among subpopulations; P-Value = Significant level for F-statistics (significance is $P \leq 0.0500$).

Table S4 Characteristics of *P. tomentosa* hybrids

Cross parent ID		Provenance	Geographical names	Geographical distance	Genetic distance
♀	♂				
	1516	Heibei	Handan	262.85	0.3874
	1106		Zhangjiakou	710.71	0.3485
	2-3	Shandong	Heze	92.78	0.5141
	3-85-1	Henan	Zhumadian	217.3	0.6422
	3353		Kaifeng	131.14	0.5991
	3709		Luohe	177.97	0.5141
	3807		Zhumadian	205.3	0.3874
	3-97-3		Nanyang	332.11	0.6132
3243	5018	Shananxi	Hanzhong	815.03	0.3874
	5013		Hanzhong	755.72	0.3874
	5019		Hanzhong	787.19	0.5051
	5074		Hanzhong	798.95	0.3485
	5066		Hanzhong	770.53	0.4302
	5084		Baoji	747.46	0.4829
	5120		Xianyang	633.97	0.5565
	8210	Anhui	Suzhou	147.64	0.3485
	8212		Suzhou	150.38	0.3961
	1516	Heibei	Handan	796.43	0.1972
	1106		Zhangjiakou	1108.09	0.2189
	2-3	Shandong	Heze	816.76	0.4669
	3-85-1	Henan	Zhumadian	617.3	0.5104
	3353	Henan	Kaifeng	703.87	0.4963
	3709		Luohe	667.93	0.3979
	3807		Zhumadian	650.98	0.2343
	3-97-3		Nanyang	513.78	0.5104
5088	5018	Shaxi	Xianyang	115.77	0.1972
	5013		Xianyang	95.72	0.1972
	5019		Xianyang	87.19	0.4264
	5074		Xianyang	98.95	0.2189
	5066		Xianyang	70.53	0.3232
	5084		Xianyang	147.46	0.6470
	5120		Xianyang	210.72	0.0764
	8210	Anhui	Suzhou	908.64	0.2189
	8212		Suzhou	944.95	0.2002

Table S5 SSR genetic distance between females with high fertility and males with large pollen quantities in *Populus tomentosa*

Male	Female								
	T-F-1	T-F-2	T-F-3	T-F-4	T-F-5	T-F-6	T-F-7	T-F-8	T-F-9
T-M-1	0.3013	0.361	0.315	0.3013	0.2926	0.3814	0.5555	0.3013	0.3273
T-M-2	0.4801	0.4366	0.4938	0.4801	0.4756	0.5689	0.7697	0.4801	0.5148
T-M-3	0.3013	0.361	0.315	0.3013	0.2926	0.3612	0.5302	0.3013	0.3273
T-M-4	0.3211	0.3395	0.3348	0.3211	0.3128	0.402	0.5555	0.3211	0.3479
T-M-5	0.4407	0.2266	0.4089	0.4177	0.4121	0.4807	0.4989	0.4177	0.4036
T-M-6	0.326	0.3456	0.3397	0.326	0.318	0.4076	0.5661	0.326	0.3535
T-M-7	0.3413	0.361	0.355	0.3413	0.3334	0.425	0.5815	0.3413	0.3689
T-M-8	0.3361	0.3979	0.3498	0.3361	0.3277	0.4169	0.5451	0.3361	0.3628
T-M-9	0.326	0.3456	0.3397	0.326	0.318	0.4076	0.5661	0.326	0.3535
T-M-10	0.3891	0.3901	0.4028	0.3891	0.3825	0.4292	0.5928	0.3891	0.4195
T-M-11	0.5802	0.4854	0.5426	0.5542	0.5256	0.6202	0.3119	0.5542	0.5401
T-M-12	0.5104	0.3979	0.5241	0.5104	0.4818	0.5745	0.6231	0.5104	0.4963
T-M-13	0.4111	0.3676	0.4248	0.4111	0.405	0.5201	0.5401	0.4111	0.4425
T-M-14	0.7679	0.4948	0.7816	0.7679	0.7721	0.9838	0.6615	0.7679	0.7538
T-M-15	0.3466	0.3676	0.3603	0.3466	0.339	0.4292	0.5401	0.3466	0.375
T-M-16	0.4413	0.361	0.355	0.3413	0.3334	0.425	0.5815	0.3413	0.3689
T-M-17	0.672	0.3184	0.6269	0.6422	0.6435	0.7121	0.658	0.6422	0.6281
T-M-18	0.3211	0.3395	0.3348	0.3211	0.5128	0.402	0.5555	0.3211	0.3479
T-M-19	0.6302	0.2919	0.5883	0.602	0.6016	0.6702	0.6749	0.602	0.5879
T-M-20	0.1573	0.5107	0.1539	0.1402	0.1638	0.2688	0.6483	0.1402	0.1262
T-M-21	0.3466	0.3676	0.4603	0.4466	0.339	0.4292	0.5401	0.3466	0.375
T-M-22	0.3163	0.3544	0.33	0.3163	0.3075	0.4269	0.5451	0.3163	0.3422
T-M-23	0.5383	0.2872	0.502	0.513	0.5098	0.5784	0.5769	0.513	0.4989
T-M-24	0.3211	0.3395	0.3348	0.3211	0.3128	0.402	0.5555	0.3211	0.3479
T-M-25	0.3211	0.3395	0.3348	0.3211	0.3128	0.402	0.5555	0.3211	0.3479
T-M-26	0.3058	0.3676	0.3195	0.3058	0.2974	0.3866	0.5928	0.3058	0.3325
T-M-27	0.3361	0.3979	0.33	0.3361	0.3483	0.4595	0.6786	0.3361	0.0322
T-M-28	0.3211	0.3395	0.3348	0.3211	0.3128	0.402	0.5555	0.3211	0.3479
T-M-29	0.3211	0.3395	0.3348	0.3211	0.3128	0.402	0.5555	0.3211	0.3479
T-M-30	0.4642	0.4448	0.4779	0.4642	0.4597	0.553	0.6325	0.4642	0.4989
T-M-31	0.8533	0.5156	0.867	0.8533	0.8988	0.9674	0.6749	0.8533	0.8392
T-M-32	0.4045	0.3395	0.4182	0.4045	0.3979	0.489	0.6637	0.4045	0.3479
T-M-33	0.513	0.3742	0.4779	0.4883	0.4844	0.5784	0.4501	0.4883	0.4742
T-M-34	0.4719	0.4284	0.4856	0.4719	0.468	0.6146	0.6451	0.4719	0.5097
T-M-35	0.689	0.4043	0.7027	0.689	0.6912	0.7915	0.7702	0.689	0.6749
T-M-36	0.3413	0.361	0.355	0.3413	0.3334	0.425	0.5815	0.3413	0.3689

Continued **table S5**

Male	Female								
	T-F-1	T-F-2	T-F-3	T-F-4	T-F-5	T-F-6	T-F-7	T-F-8	T-F-9
T-M-37	0.455	0.1932	0.4211	0.4309	0.4264	0.495	0.4909	0.4309	0.4568
T-M-38	0.4336	0.3676	0.4473	0.4336	0.428	0.5201	0.5401	0.4336	0.466
T-M-39	0.3211	0.3395	0.3348	0.3211	0.3128	0.402	0.5555	0.3211	0.3479
T-M-40	0.3211	0.3395	0.3348	0.3211	0.3128	0.402	0.5555	0.3211	0.3479
T-M-41	0.602	0.4531	0.6157	0.602	0.5734	**0.729**	0.7057	0.602	0.5879
T-M-42	0.3211	0.3395	0.3348	0.3211	0.3128	0.402	0.5555	0.3211	0.3479
T-M-43	0.4801	0.4854	0.4938	0.4801	0.4515	0.5689	**0.738**	0.4801	0.5148
T-M-44	0.6466	0.5208	0.6603	0.6466	0.618	**0.7454**	0.5502	0.6466	0.6913
T-M-45	**0.8346**	0.5003	**0.7816**	**0.7679**	**0.7721**	**0.9097**	**0.7221**	0.7679	0.8205
T-M-46	0.3891	0.4131	0.425	0.3891	0.3825	0.4736	0.5401	0.3891	0.4195
T-M-47	0.4639	0.4434	0.4776	0.4639	0.4353	0.5504	0.5704	0.4639	0.4963
T-M-48	0.4045	0.3395	0.4182	0.4045	0.3979	0.489	0.5555	0.4045	0.4349
T-M-49	0.4336	0.4607	0.4473	0.4336	0.428	0.5201	0.5928	0.4336	0.466
T-M-50	0.3466	0.3456	0.3603	0.3466	0.339	0.4292	0.5928	0.3466	0.375
T-M-51	0.3163	0.3759	0.37	0.3163	0.3075	0.438	0.6231	0.3163	0.3422
T-M-52	0.4045	0.4285	0.4182	0.4045	0.3979	0.489	0.5555	0.4045	0.4349
T-M-53	0.3466	0.3676	0.3603	0.3466	0.339	0.4292	0.5401	0.3466	0.375
T-M-54	0.5576	0.2538	0.5447	0.5576	0.5565	0.6251	0.6281	0.5576	0.5436
T-M-55	0.513	0.3742	0.4779	0.4883	0.5098	0.6584	0.6043	0.4883	0.5243
T-M-56	0.3413	0.361	0.355	0.3413	0.3334	0.425	0.5815	0.3413	0.3689
T-M-57	0.3413	0.361	0.355	0.3413	0.3334	0.425	0.5815	0.3413	0.3689
T-M-58	0.3676	0.4366	0.3813	0.3676	0.3606	0.4511	**0.738**	0.3676	0.3535
T-M-59	0.455	0.3638	0.4211	0.4309	0.4264	0.495	**0.7872**	0.4309	0.4168
T-M-60	0.326	0.3456	0.3397	0.326	0.318	0.4076	0.5661	0.326	0.3535
T-M-61	0.6184	0.2464	0.578	0.591	0.5624	**0.7762**	0.5502	0.591	0.5769
T-M-62	0.3058	0.4131	0.3195	0.3058	0.2974	0.366	0.5928	0.3058	0.3325
T-M-63	0.602	0.3134	0.6157	0.602	0.6016	0.6702	0.6451	0.602	0.5879
T-M-64	0.4339	0.4349	0.4476	0.4339	0.4054	0.5184	0.5596	0.4339	0.4643
T-M-65	0.3013	0.361	0.315	0.3013	0.2926	0.3814	0.5555	0.3013	0.3273
T-M-66	0.4797	0.5285	0.4687	0.4797	0.4764	0.545	0.5169	0.4797	0.4656
T-M-67	0.4414	0.4434	0.4551	0.4414	0.3277	0.4814	0.6787	0.4414	0.4728
T-M-68	0.4566	0.4854	0.4703	0.4556	0.4515	0.5689	0.7072	0.4566	0.4901

Note: The genetic distances between the parents of the selected hybrid combinations are shown in bold type.

Table S6 SSR genetic distance between females with high fertility and males with large pollen quantities in *Populus tomentosa*

Male	Female								
	T-F-10	T-F-11	T-F-12	T-F-13	T-F-14	T-F-15	T-F-16	T-F-17	T-F-18
T-M-1	0.1153	0.3185	0.2678	0.3273	0.2956	0.3013	0.3211	0.3620	0.2040
T-M-2	0.2112	0.3901	0.4425	0.5148	0.4703	0.4801	0.5042	0.5542	0.2877
T-M-3	0.1153	0.3185	0.2678	0.3273	0.2956	0.3013	0.3211	0.3620	0.2424
T-M-4	0.1330	0.2978	0.2872	0.3479	0.3150	0.3211	0.3413	0.3830	0.2230
T-M-5	0.2353	0.2666	0.4266	0.4036	0.4089	0.4177	0.4177	0.4642	0.3818
T-M-6	0.1357	0.3031	0.2971	0.3535	0.3195	0.3260	0.3466	0.3891	0.2271
T-M-7	0.1511	0.3185	0.3071	0.3689	0.3348	0.3413	0.3620	0.4045	0.2424
T-M-8	0.1480	0.2926	0.3022	0.3628	0.3300	0.3361	0.3563	0.3979	0.2380
T-M-9	0.1357	0.3031	0.2917	0.3535	0.3195	0.3260	0.3466	0.3891	0.2271
T-M-10	0.1727	0.3456	0.3535	0.4195	0.3813	0.3891	0.4111	0.4111	0.2469
T-M-11	0.4541	0.4607	0.5661	0.5401	0.5426	0.5542	0.5542	0.5802	0.4212
T-M-12	0.3232	0.4204	0.4728	0.4963	0.5006	0.5104	0.4869	0.5104	0.4756
T-M-13	0.1918	0.3241	0.375	0.4425	0.4248	0.4111	0.4336	0.4801	0.3302
T-M-14	0.4636	0.4948	**0.7221**	**0.7538**	**0.7816**	**0.7679**	**0.7362**	**0.9061**	0.6166
T-M-15	0.1540	0.3241	0.3119	0.3750	0.3397	0.3466	0.3676	0.4111	0.2469
T-M-16	0.1511	0.3185	0.3071	0.3689	0.3348	0.3413	0.3620	0.4045	0.2424
T-M-17	0.3802	0.3638	0.6580	0.6281	0.6269	0.6422	0.6422	0.7028	0.5261
T-M-18	0.1330	0.2978	0.2872	0.3479	0.3150	0.3211	0.3413	0.3830	0.2230
T-M-19	0.3731	0.3353	0.6161	0.5879	0.5883	0.6020	0.6020	0.6591	0.4890
T-M-20	0.4794	0.4854	0.1783	0.4262	0.4539	0.1402	0.1402	0.2665	0.2469
T-M-21	0.1918	0.3241	0.3119	0.3750	0.3397	0.3466	0.3676	0.4111	0.2469
T-M-22	0.1303	0.3128	0.2828	0.3422	0.3105	0.3163	0.3361	0.3769	0.2380
T-M-23	0.3429	0.3298	0.5243	0.4989	0.5020	0.5130	0.5130	0.5643	0.4053
T-M-24	0.1330	0.2978	0.2872	0.3479	0.3150	0.3211	0.3413	0.3830	0.2230
T-M-25	0.1330	0.2978	0.2872	0.3479	0.5150	0.3211	0.3413	0.3830	0.2230
T-M-26	0.1540	0.3241	0.2524	0.3325	0.2996	0.3058	0.3260	0.3676	0.2271
T-M-27	0.2415	0.3334	0.2637	0.3220	0.3300	0.3361	0.3163	0.4194	0.3606
T-M-28	0.1330	0.2978	0.2872	0.3479	0.3150	0.3211	0.3413	0.3830	0.2230
T-M-29	0.1511	0.2978	0.2872	0.3479	0.3150	0.3211	0.3413	0.3830	0.2424
T-M-30	0.2559	0.3972	0.4266	0.4989	0.4544	0.4642	0.4883	0.5383	0.3588
T-M-31	0.5540	0.5156	**0.8041**	**0.8392**	**0.8670**	**0.8533**	**0.8182**	**0.9666**	0.6926
T-M-32	0.2070	0.3830	0.3689	0.3479	0.3967	0.4045	0.3413	0.4720	0.3031
T-M-33	0.4136	0.3517	0.4989	0.4742	0.4779	0.4883	0.4883	0.5383	0.4053
T-M-34	0.2606	0.3808	0.4338	0.5079	0.4856	0.4719	0.4966	0.5746	0.3889
T-M-35	0.3495	0.4043	0.6451	0.6749	0.7027	0.6890	0.6591	**0.7515**	0.5157
T-M-36	0.1511	0.3185	0.3071	0.3689	0.3348	0.3413	0.3620	0.4045	0.2424

Continued **table S6**

Male	Female								
	T-F-10	T-F-11	T-F-12	T-F-13	T-F-14	T-F-15	T-F-16	T-F-17	T-F-18
T-M-37	0.2651	0.2332	0.4409	0.4168	0.4211	0.4309	0.4309	0.4797	0.3720
T-M-38	0.4512	0.3241	0.397	0.4660	0.4248	0.4336	0.4566	0.5042	0.3522
T-M-39	0.4330	0.2978	0.2872	0.3479	0.3150	0.3211	0.3413	0.3830	0.2230
T-M-40	0.1330	0.2978	0.2872	0.3479	0.3150	0.3211	0.3413	0.3830	0.2230
T-M-41	0.3495	0.4531	0.5605	0.5879	0.5883	0.6020	0.5746	0.6890	0.3424
T-M-42	0.1330	0.2978	0.2872	0.3479	0.3150	0.3211	0.3413	0.3838	0.2230
T-M-43	0.3363	0.4366	0.4195	0.5148	**0.7703**	0.4801	0.5042	0.5289	**0.7252**
T-M-44	0.3659	0.4695	0.6043	0.6913	0.6321	0.6466	0.6755	0.7054	0.4294
T-M-45	0.5598	0.5611	0.6913	**0.8205**	**0.7816**	**0.7679**	**0.7679**	**0.9061**	**0.8108**
T-M-46	0.1918	0.3676	0.3535	0.4195	0.3813	0.3891	0.4111	0.4566	0.2877
T-M-47	0.2221	0.3979	0.4273	0.4963	0.4551	0.4639	0.4869	0.5104	0.3180
T-M-48	0.2072	0.2978	0.3689	0.4349	0.3967	0.4045	0.4265	0.472	0.3031
T-M-49	0.2310	0.4131	0.3970	0.466	0.4248	0.4336	0.4566	0.5042	0.2877
T-M-50	0.1357	0.3031	0.3119	0.3750	0.3397	0.3466	0.3676	0.4111	0.2469
T-M-51	0.1843	0.3334	0.2828	0.3422	0.3105	0.3163	0.3361	0.4194	0.2189
T-M-52	0.2266	0.3830	0.3689	0.4349	0.3967	0.4045	0.4265	0.4720	0.3241
T-M-53	0.1540	0.3241	0.3119	0.3750	0.3397	0.3466	0.3676	0.4111	0.2469
T-M-54	0.2436	0.2538	0.5169	0.5436	0.5447	0.5576	0.5310	0.6132	0.4721
T-M-55	0.4636	0.3742	0.5502	0.5243	0.5020	0.4883	0.5383	0.6184	0.5054
T-M-56	0.1330	0.3185	0.3071	0.3689	0.3348	0.3413	0.3620	0.4045	0.2424
T-M-57	0.1551	0.3185	0.3071	0.3689	0.3348	0.3413	0.3620	0.4045	0.2424
T-M-58	0.2512	0.4366	0.2917	0.3535	0.3603	0.4676	0.3466	0.4336	0.3087
T-M-59	0.1818	0.4115	0.4409	0.4168	0.4211	0.4309	0.4309	0.4797	0.3485
T-M-60	0.1540	0.3031	0.2917	0.3535	0.3195	0.3260	0.3466	0.3891	0.2469
T-M-61	0.3659	0.2872	0.6043	0.5769	0.6047	0.5910	0.5910	0.6755	0.4541
T-M-62	0.1540	0.3676	0.2524	0.3325	0.2996	0.5058	0.3260	0.3676	0.2671
T-M-63	0.2821	0.3134	0.5605	0.5879	0.5883	0.6020	0.5746	0.6890	0.5157
T-M-64	0.2366	0.3904	0.3983	0.4643	0.4261	0.4339	0.4559	0.4784	0.3325
T-M-65	0.1153	0.3185	0.2678	0.3273	0.2956	0.3013	0.3211	0.362	0.2040
T-M-66	0.2871	0.5415	0.4409	0.4656	0.4687	0.4797	0.4550	0.5310	0.3961
T-M-67	0.3232	0.4204	0.4054	0.4728	0.4331	0.4414	0.4639	0.3563	0.4515
T-M-68	0.2310	0.4366	0.4195	0.4901	0.4703	0.4566	0.4801	0.5289	0.2671

Note: The genetic distances between the parents of the selected hybrid combinations are shown in bold type.

Table S7 The distribution table of the high combining ability hybrid combinations based on the genetic distance prediction

**Male**	**Female**														
	**T-F-1**	**T-F-3**	**T-F-4**	**T-F-5**	**T-F-6**	**T-F-7**	**T-F-8**	**T-F-9**	**T-F-12**	**T-F-13**	**T-F-14**	**T-F-15**	**T-F-16**	**T-F-17**	**T-F-18**
T-M-2						○									
T-M-14	○	○	○	○	○		○	○	○	○	○	○	○	○	
T-M-17					○										
T-M-31	○	○	○	○	○		○	○	○	○	○	○	○	○	
T-M-35					○	○								○	
T-M-41					○										
T-M-43						○					○				
T-M-44					○										
T-M-45	○	○	○	○	○	○	○	○		○	○	○	○	○	○
T-M-58						○									
T-M-59						○									
T-M-61					○										

○ represents high specific combining ability hybridization combinations based on genetic distance prediction

Table S8 High combining ability hybrid combinations based on half-sib progeny paternal identification

	Highly fertile female parent																	
Male	T-F-1	T-F-2	T-F-3	T-F-4	T-F-5	T-F-6	T-F-7	T-F-8	T-F-9	T-F-10	T-F-11	T-F-12	T-F-13	T-F-14	T-F-15	T-F-16	T-F-17	T-F-18
T-M-2									□									
T-M-14						□									□			
T-M-16	□																	
T-M-20													□	□				□
T-M-21			□	□														
T-M-22						□												
T-M-23		□																
T-M-25	□													□				
T-M-27									□									
T-M-31											□							
T-M-33														□				
T-M-34	□		□			□												
T-M-35													□					
T-M-37									□									
T-M-38								□										
T-M-39										□								

Continued **table S8**

Male	Highly fertile female parent																	
	T-F-1	T-F-2	T-F-3	T-F-4	T-F-5	T-F-6	T-F-7	T-F-8	T-F-9	T-F-10	T-F-11	T-F-12	T-F-13	T-F-14	T-F-15	T-F-16	T-F-17	T-F-18
T-M-41															□			
T-M-43														□				□
T-M-62															□			
T-M-66	□					□						□						
T-M-67			□															
T-M-68															□			
T-M-69					□													
T-M-44														□	□			
T-M-45	□		□			□	□	□	□					□	□	□		□
T-M-46			□															
T-M-58															□			
T-M-59															□			
T-M-61						□												

Note: □ represents hybridization combinations based on male parent identification.

Table S9 Information on the full-sib progeny group in *P. tomentosa*

Female	Male	Seed number	Number of seedlings	Germination rate	Number of surviving seedlings	Survival rate
T-F-15	T-M-2	4998	1424	28.5	995	69.9
	T-M-14	1906	192	10.1	137	71.4
	T-M-41	3418	342	10.0	247	72.2
T-F-14	T-M-45	2800	170	6.1	137	80.6
	T-M-27	3654	1106	31.0	803	72.6
	T-M-43	5310	1584	29.8	1210	76.4
T-F-18	T-M-45	6810	252	3.7	205	81.3
	T-M-43	5124	345	6.7	250	72.5
Total (Mean)		34020	5415	15.9	3984	73.6
